# A Chinese boy with episodic ataxia and low CSF glucose

**DOI:** 10.1186/1687-9856-2013-S1-P173

**Published:** 2013-10-03

**Authors:** Kwai-fun Huen

**Affiliations:** 1 Chief of Service and Consultant, Department of Paediatrics & Adolescent Medicine, Tseung Kwan O Hospital, Hong Kong

## 

A 3-year-8-month-old Chinese boy presented with acute exacerbation of ataxia precipitated by a viral illness. Past health revealed a clumsy boy with unsteady gait all along associated with moderate language and gross and fine motor delay but relatively preserved cognitive function. He was born normally at full term to non-consanguinous parents. There was no history of perinatal asphyxia or convulsion. The movement problem was paroxysmal, worse in early morning and during sick days. No history of any drugs was taken. Examination revealed a conscious boy with normal growth and head size. He had cerebellar ataxia with wide-based unsteady gait, truncal ataxia, intentional tremor and horizontal nystagmus. Cranial nerves, muscle tone, power and reflexes were normal. Gross sensation and hearing were normal. No skin rash, telangiectasia or neurocutaneous stigmata was noted. MRI was normal. CSF showed a lowish glucose of 1.9 mmol/l at a blood glucose of 4.8 mmol/l (CSF/blood glucose ratio 0.4), lactate and other biochemistries were normal, viral and bacterial cultures were negative. In view of the episodic nature with acute exacerbation during viral illnesses, congenital metabolic disease was highly suspected. Metabolic screening including plasma and urine amino acids, urine organic acids, plasma carnitine /acylcarnitine, ammonia, very-long-chain-fatty-acid, lyzosomal enzymes, transferrin, copper, ceruloplasmin and pre and post meal lactate, pyruvate, betahydroxybutyrate, acetoacete and glucose were all normal. Glucose transporter defect was suspected. However, common mutation SLC2A1 gene (1p35-31.3) for glucose transport protein (GLUT1) deficiency was not detected. Erythrocyte uptake test showed decreased glucose uptake. The clinical features together with low CSF glucose and decreased erythrocyte uptake were compatible with GLUT1 deficiency. Ketogenic diet with modified Atkins diet was started. Ataxia showed marked improvement. The diet was well-tolerated. No adverse effect was noted.

GLUT1 deficiency commonly presents with infantile epileptic encephalopathy, movement disorder, progressive microcephaly and psychomotor retardation. It is inherited as an autosomal recessive disorder, 70-80% associated with SLC2A1 gene (1p35-31.3) mutation. The prognosis is good with early diagnosis and treatment with ketogenic diet (KD). Recent research reported similar efficacy to classical KD with lower ketogenic ratios (2:1 or 1:1), such as medium-chain-triglyceride oil diet, modified Atkins diet, and low-glycemic-index treatment. These allow better tolerability and less adverse effects. Further research makes KD more widely available, effective and safer.

